# The Role of Diet in Shaping Gut Microbiota and Its Impact on Host Metabolic Regulation

**DOI:** 10.3390/ijms27062768

**Published:** 2026-03-18

**Authors:** Andrea Esthefania Hernández-Valles, Gabriela Martínez-Machado, Litzy Yazmin Alvarado-Mata, Carlos Lopez-Ortiz, Padma Nimmakayala, Nagamani Balagurusamy, Umesh K. Reddy

**Affiliations:** 1 Department of Biology, Gus R. Douglass Institute, West Virginia State University, Institute, WV 25112, USA; andrea.valles@wvstateu.edu (A.E.H.-V.); gabriela.machado@wvstateu.edu (G.M.-M.); litzy.mata@wvstateu.edu (L.Y.A.-M.); carlos.ortiz@wvstateu.edu (C.L.-O.); padma@wvstateu.edu (P.N.); 2 Laboratorio de Biorremediación, Facultad de Ciencias Biológicas, Universidad Autónoma de Coahuila, Torreón 27000, Coahuila, Mexico

**Keywords:** gut microbiome, dietary modulation, host metabolism, microbial metabolites, dysbiosis

## Abstract

Diet is a key modulator of the gut microbiota, thereby influencing host physiology. Microbial colonization begins early in life, influenced by maternal sources, mode of birth, diet, and environmental exposures, and stabilizes into an adult-like microbiome during early childhood. This maturation yields a microbial ecosystem dominated by *Firmicutes* and *Bacteroidetes* that contributes to host physiological homeostasis. Gut microorganisms function as an integrated metabolic system that transforms dietary substrates into bioactive metabolites, including short-chain fatty acids (SCFAs), amino acid-derived compounds, and microbial lipids. These metabolites regulate glucose and lipid metabolism, intestinal barrier integrity, and immune modulation. Although many metabolic functions are conserved, their activity is shaped by diet, microbial cross-feeding, and local intestinal conditions, enabling functional specialization within the gut. Disruption of this system, known as dysbiosis, is associated with alterations in microbial diversity and metabolic output that have been linked to metabolic diseases, including obesity and related disorders. Evidence from experimental models and observational studies suggests that these associations may involve interconnected inflammatory and metabolic mechanisms, such as impaired intestinal barrier function, low-grade inflammation, and altered dietary energy harvest; however, causal relationships in humans remain incompletely understood. Beyond peripheral effects, the gut microbiome influences host metabolism via the gut–brain axis, a bidirectional network that integrates neural, endocrine, immune, and metabolic signaling. Microbiota-derived metabolites and gut hormone modulation contribute to appetite regulation, energy balance, and glucose homeostasis, while central neuroendocrine signaling can reciprocally shape the intestinal microbial niche. Collectively, these findings highlight the gut microbiome as a central regulator of host metabolism, whose disruption may contribute to the development of metabolic disease.

## 1. Introduction

Dietary intake supplies the host with essential macronutrients and provides substrates that shape the metabolic activity of the gut microbiota [1]. Through the biotransformation of dietary carbohydrates, proteins, lipids, and phytochemicals, intestinal microorganisms generate a diverse range of metabolites that act locally within the gut and systemically throughout the host. These diet–microbiota interactions play a central role in regulating glucose and lipid homeostasis, immune function, epithelial barrier integrity, and energy balance [2].

The metabolic contribution of the gut microbiota reflects the limited enzymatic capacity of the human genome to degrade complex dietary components. In particular, non-digestible carbohydrates and many phytochemicals escape host digestion. They are metabolized by microbial enzymes, including carbohydrate-active enzymes such as glycoside hydrolases, thereby converting complex substrates into bioactive compounds [3,4]. This metabolic complementarity underlies host–microbe co-metabolism and enables the gut microbiota to function as an essential metabolic extension of the host [5]. Beyond carbohydrates, dietary proteins and lipids also significantly influence the structure, composition, and functionality of the gut microbiota [6]. Diets rich in animal protein, saturated fats, refined carbohydrates, and salt have been shown to promote the growth of pathogenic bacteria and reduce microbial diversity [7].

In addition, microbiota–host interactions involve bidirectional communication mediated by microbial metabolites and signaling molecules that influence both epithelial responses and microbial activity [8]. The gut microbiota shapes the host metabolic phenotype by producing signaling molecules that regulate host metabolism, bile acid homeostasis, gut permeability, and hormone release [1,5], and disruption of this balance, known as dysbiosis, can impair this communication and is linked to metabolic disorders such as obesity, insulin resistance, and type 2 diabetes [9]. The gut–brain axis further links microbial metabolism to appetite regulation and energy balance, thereby reinforcing the microbiota’s role in the development of metabolic disease [10].

While early microbiome research focused on taxonomic composition, accumulating evidence indicates that microbial metabolic function, rather than community structure alone, determines metabolic outcomes [6]. Identical dietary exposures can yield divergent physiological responses depending on microbial functional capacity, substrate availability, and ecological interactions such as metabolite cross-feeding [11]. Accordingly, understanding how diet shapes microbial metabolism and how these metabolic outputs regulate host physiology is essential for elucidating the mechanisms linking nutrition to metabolic health and disease [9]. In this review, we synthesize current knowledge on how diet–microbiota interactions shape host physiology through functional microbial metabolism. Specifically, we focus on key microbial metabolic outputs, including SCFAs, amino acid-derived metabolites, and microbial lipids, and discuss how these metabolites integrate dietary inputs with host metabolic regulation and gut–brain signaling pathways. By emphasizing microbial metabolic functions and their dietary determinants, this review highlights emerging mechanisms by which diet-driven microbial activity influences host health and may inform future strategies to modulate microbial metabolism via targeted dietary interventions.

## 2. Microbial Establishment Across the Human GI Tract

The gastrointestinal (GI) tract is a spatially structured ecosystem in which anatomical and physicochemical gradients shape microbial colonization and metabolic activity, thereby helping to preserve host homeostasis [12]. Variations in oxygen availability, pH, nutrient flux, and host antimicrobial defenses generate distinct microbial niches along the gut, selectively favoring microorganisms with specialized metabolic strategies [13,14]. Oxygen availability decreases from the epithelial surface toward the intestinal lumen and from proximal to distal regions of the GI tract. This gradient supports facultative anaerobes in the upper gut and obligate anaerobes in the colon, where fermentative metabolism predominates [14,15]. Maintaining luminal hypoxia is essential for the expansion of anaerobic bacteria that produce SCFAs, particularly butyrate, which plays a key role in intestinal and systemic metabolic regulation [13,16].

In parallel, microbial density increases markedly in the distal intestine, where undigested dietary components, especially complex carbohydrates, serve as substrates for fermentation [12]. The colonic microbiota is dominated by *Firmicutes* and *Bacteroidetes*, with additional contributions from *Actinobacteria* and *Verrucomicrobia*, collectively generating metabolites that influence host energy metabolism, immune function, and epithelial integrity [17,18]. Microbial establishment is also shaped by developmental timing. Early-life colonization originates from maternal sources and is influenced by birth mode, diet, and environmental exposures [19,20]. During infancy and early childhood, the microbiome undergoes rapid functional maturation before stabilizing into an adult-like configuration [21,22]. Microbial communities established during this period exert long-lasting effects on metabolic capacity, highlighting early diet as a critical determinant of lifelong metabolic health [23].

## 3. Regulation of Host Metabolism by Diet–Microbiota Interactions

**Figure 1:**
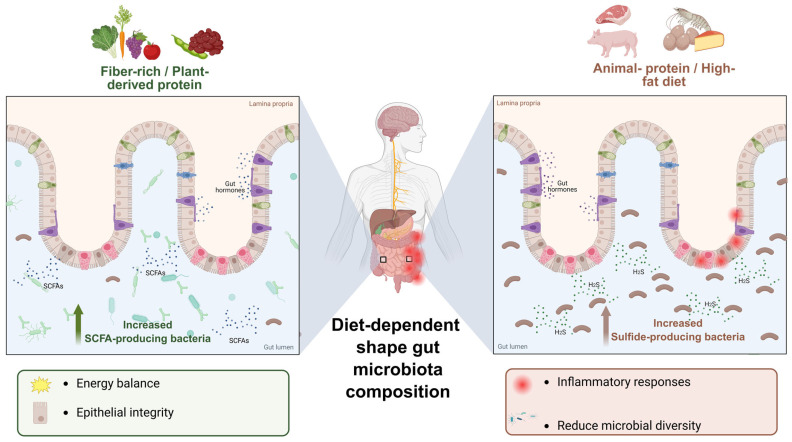
Diet-dependent modulation of gut microbiota and metabolic output. A fiber-rich, plant-based diet enriches SCFA-producing bacteria and increases luminal SCFAs, supporting epithelial integrity and metabolic homeostasis. In contrast, animal protein- and fat-rich diets promote sulfide-producing and proteolytic bacteria and elevate H_2_S, which is associated with inflammation, barrier stress, and reduced microbial diversity.

Microbial communities are shaped through various interactions ranging from competition to mutualism. In the human gut microbiome, the cross-feeding of central metabolites, including sugars, electron donors and acceptors, and essential nutrients, such as amino acids, cofactors, and vitamins, exchanged between microbial groups and the host drives the formation of gut commensal communities that are resilient to invasion and maintain ecological stability [11]. Gut microbiota-derived metabolites have been shown to regulate different host processes, including glucose and lipid metabolism, energy consumption, and the immune system. In addition, microbiota-derived metabolites act as key regulators of epigenetic mechanisms, including DNA methylation, RNA modifications, and histone post-translational modifications, in intestinal cells, thereby modulating gene expression involved in epithelial integrity, immune tolerance, and metabolic homeostasis [24]. Together, these interactions define the gut microbiota as an integrated metabolic system that shapes host metabolism [6]. Dietary composition is a primary driver of these microbial metabolic interactions, and nutritional content varies across dietary patterns, with fiber-rich diets associated with the expansion of SCFA-producing bacteria, which maintain epithelial integrity, gut hormone signaling, and energy balance. In contrast, animal-protein diets are linked to the expansion of sulfide-producing bacteria, leading to increased hydrogen sulfide (H_2_S) production, which may be associated with inflammatory responses, reduced microbial diversity, and disruption of intestinal homeostasis (Figure 1) [25].

The intestinal environment is shaped by the small intestine’s structure, which enables efficient nutrient absorption while also providing an ecosystem for diverse microorganisms [26]. In this context, gut microbiota-derived metabolites, including SCFAs and amino acid-derived metabolites, play a crucial role in host homeostasis. These metabolites can enter the systemic circulation and modulate organ-specific functions, thereby impacting overall host physiology. In addition, they influence the gut ecosystem by modulating the activity, growth, and competitiveness of other microbial populations, including potential pathogens [27].

### 3.1. Diet and Microbial Metabolism

Among the multiple host and environmental factors influencing the gut microbiota, diet and its components represent the primary regulators of microbial composition and activity [28]. Diet is a major driver of microbial metabolic activity, providing substrates that gut microorganisms transform into bioactive metabolites (Table 1). Dietary substrates that escape absorption in the small intestine, such as inulin, pectin, fructooligosaccharides, and proteins, are metabolized by gut microorganisms into smaller molecules, including sugars, fatty acids, and amino acids. While most dietary lipids are absorbed in the small intestine before reaching the colon, gut bacteria can metabolize certain lipid-derived components, such as glycerol and phospholipids. Gut bacteria can also modify specific polyunsaturated fatty acids (e.g., linoleic acid) into bioactive microbial metabolites. Through these metabolic activities, the gut microbiota contributes to host metabolic regulation, particularly by producing metabolites that influence glucose homeostasis and lipid-related signaling pathways [29].

### 3.2. SCFA-Mediated Metabolic Regulation

**Figure 2:**
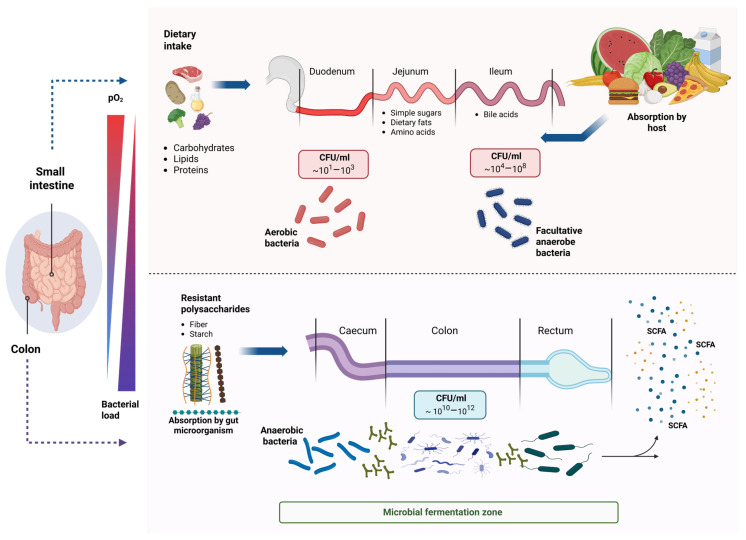
Spatial distribution of bacterial communities and metabolic specialization along the human gastrointestinal tract. The GI tract exhibits a progressive decrease in oxygen partial pressure (pO_2_), accompanied by a concomitant increase in bacterial load, from low-density, oxygenated regions of the small intestine (~10^1^ߝ10^8^ CFU/mL) to the highly anaerobic, densely colonized large intestine (~10^10^ߝ10^12^ CFU/mL). As oxygen decreases toward the large intestine, anaerobic bacteria dominate, fermenting resistant polysaccharides such as fiber and resistant starch to produce SCFAs.

Among dietary components, carbohydrates represent a major energy source for the host and gut microorganisms, and can be classified as digestible or non-digestible. Digestible carbohydrates are rapidly broken down in the small intestine and released into the bloodstream as glucose. These include monosaccharides (e.g., glucose and fructose), disaccharides (e.g., lactose and galactose), and polysaccharides, including maltodextrin and starch [37,38]. In contrast, non-digestible carbohydrates such as resistant starch and dietary fiber are not hydrolyzed by host digestive enzymes and therefore pass largely intact through the small intestine, reaching the colon, where they become substrates for microbial fermentation [39]. These compounds serve as carbon sources for specific gut microorganisms, promoting the expansion of saccharolytic taxa, including members of the phylum *Firmicutes*. Within the colonic environment, although these genome-encoded metabolic functions are conserved, their expression and activity depend on local conditions, particularly the colon’s predominantly anaerobic state. Under these conditions, fermentable substrates serve as carbon sources for specialized bacteria, leading to the production of compounds such as acetate, butyrate, and propionate. Among the metabolites generated through microbial fermentation, SCFAs are considered key mediators of host–microbiota metabolic interactions because they function as energy substrates and signaling molecules. These fermentation products support microbial cross-feeding and influence host physiology [40]. Although the relative abundance of certain phyla can modulate SCFA biosynthesis, studies indicate that production depends on metabolic pathways rather than taxonomy alone and is further shaped by diet and the intestinal environment [16]. Fermentative bacteria belonging to *Firmicutes* and *Actinobacteria* are the main responders to dietary fiber as they initiate substrate degradation (Figure 2) [25]. Mice fed a low-fiber diet exhibit higher Proteobacteria content, increased permeability, and a reduced growth rate of the inner mucus layer [41].

Given that the host absorbs most SCFAs, these functional communities act as a key regulatory system that modulates host energy metabolism and metabolic homeostasis [42]. Butyrate and propionate, in particular, have been shown to regulate host metabolism by activating intestinal glucose production through distinct molecular pathways, thereby contributing to appetite control and energy balance. Moreover, as signaling molecules, these fermentation-derived metabolites coordinate intestinal pathways involved in glucose sensing and utilization. This intestinal signaling is further integrated with peripheral metabolic responses, including enhanced insulin sensitivity, reduced hepatic gluconeogenesis, improved glucose uptake, and coordinated regulation of lipid metabolism in adipose tissue, liver, and skeletal muscle [43,44].

In addition to their metabolic effects, these metabolites also play a critical role in maintaining intestinal barrier function. Butyrate has been reported to enhance epithelial integrity by upregulating the tight junction protein claudin-1 through the modulation of key signaling pathways, including AMPK, Akt, and PKC [45,46]. Similarly, acetate has been shown to activate the NLRP3 inflammasome, leading to increased IL-18 production and improved intestinal barrier function [47].

Evidence from germ-free mouse models demonstrates that gut microbiota-derived metabolites are essential regulators of hepatic drug-metabolizing enzymes, including cytochrome P450 (CYP) enzymes. Beyond their role in energy metabolism, they also contribute to xenobiotic metabolism. Notably, butyrate modulates hepatic drug metabolism through aryl hydrocarbon receptor (AhR)-dependent pathways, thereby influencing CYP enzyme expression and activity [48].

### 3.3. Protein Fermentation

Furthermore, beyond carbohydrate-derived metabolites, gut microbiota-mediated protein fermentation plays a significant role in host metabolism. Approximately 90% of dietary protein is absorbed in the small intestine, while the remaining fraction reaches the colon, where it serves as a substrate for proteolytic bacteria. Elevated protein intake has been shown to increase microbial biomass but reduce microbial diversity at both the family and genus levels [49]. Moderate dietary protein restriction can improve gut microbiota composition and preserve intestinal barrier integrity. During homeostasis, efficient host uptake of amino acids in the small intestine makes them scarce for microbes; however, an overgrowth of amino acid-utilizing bacteria (e.g., *Clostridia*) can compete with the host, especially if dietary protein is limited, whereas severe restriction disrupts microbial diversity and increases intestinal permeability [50]. Diets rich in animal-based proteins, which are high in sulfur-containing amino acids such as methionine and cysteine, may alter gut sulfur metabolism by stimulating sulfate-reducing and sulfur-metabolizing bacteria, thereby increasing H_2_S production. While H_2_S serves as a physiological signaling molecule at low concentrations, excessive luminal accumulation may impair epithelial metabolism and promote intestinal inflammation. The effect of H_2_S depends on the balance between microbial production and host detoxification capacity; excessive accumulation may inhibit cytochrome c oxidase, compromise ATP production, and disrupt barrier integrity [51]. This shift may compromise intestinal homeostasis and metabolic health [52].

In contrast, diets rich in plant-derived protein sources have been associated with an increased abundance of beneficial genera such as *Bifidobacterium*, *Faecalibacterium*, *Eubacterium*, and *Roseburia*, which are linked to butyrate production and reduced intestinal inflammation. These dietary patterns are also associated with enrichment of *Prevotella*, increased dominance of Bacteroides, and distinct microbial profiles [38,53]. Consistently, dietary patterns characterized by high intake of animal protein, saturated fats, refined carbohydrates, and salt have been shown to promote the growth of pathogenic bacteria and reduce overall microbial diversity [7].

### 3.4. Lipid Metabolism and Signaling

Beyond protein metabolism, dietary lipids also play a critical role in shaping the composition of the gut microbiota and host metabolic responses. In animal models, consumption of a high-fat diet increases intestinal permeability and mucosal immune responses and has been associated with the development of metabolic disorders, including diabetes, obesity, and chronic inflammation [54]. In this context, dietary fats serve not only as nutrients but also as substrates for microbial metabolism. Intestinal bacteria can transform dietary lipids into metabolites that cannot be synthesized by the mammalian host, such as conjugated linoleic acids (CLA), which have been shown to exert anti-inflammatory effects through the activation of PPARγ and the induction of trefoil factor 3 (TFF3) [55,56]. Furthermore, specific bacterial taxa, including *Lactobacillus, Roseburia,* and *Bifidobacterium*, actively participate in dietary lipid metabolism by transforming polyunsaturated fatty acids into less toxic derivatives as a detoxification strategy [57].

Moreover, microbiota-derived lipids act as signaling molecules recognized by host pattern recognition receptors, including Toll-like receptors (TLRs), NOD-like receptors (NLRs), C-type lectin receptors (CLRs), and G-protein-coupled receptors (GPCRs). Consistent with this, germ-free mouse models exhibit marked alterations in lipid profiles, underscoring the essential role of the gut microbiota in lipid metabolism and systemic metabolic homeostasis [58].

## 4. Metabolic Disease Generated by Disruption of the Microbiome

Metabolic homeostasis in the host is strongly influenced by microbial-derived functions that regulate energy balance, glucose homeostasis, lipid metabolism, and immune responses. Notably, the gut microbiota represents the first site of interaction with xenobiotic compounds, such as environmental pollutants and therapeutic drugs, thereby influencing their biotransformation and bioavailability [59]. Through these processes, microbial metabolism modulates host exposure to external chemical stressors, linking environmental factors and metabolic regulation.

**Figure 3:**
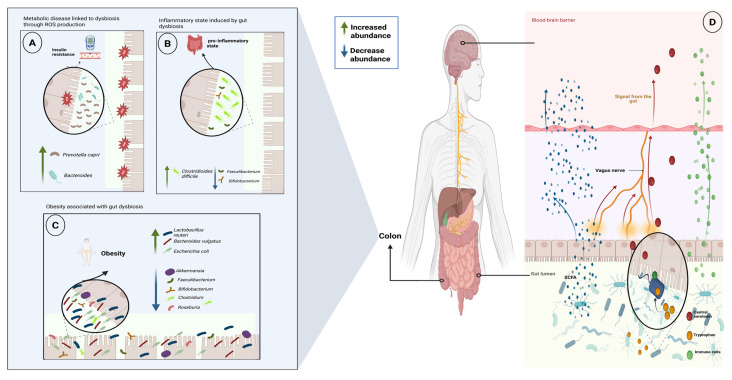
Dysbiosis, metabolic disease, and gut–brain axis integration. (**A**) Dysbiosis is associated with increased oxidative stress and may contribute to insulin resistance, particularly under high-fat dietary conditions. (**B**) Microbial imbalance, characterized by expansion of pro-inflammatory taxa and loss of beneficial SCFA-producing bacteria, may contribute to chronic low-grade inflammation. (**C**) Obesity-associated dysbiosis is often associated with reduced microbial diversity and depletion of metabolically protective genera. (**D**) Microbial metabolites, including SCFAs and tryptophan-derived compounds, can signal through the gut–brain axis, influencing appetite, gut motility, and host metabolic regulation.

Disruption of the gut microbiome, commonly referred to as dysbiosis, is associated with alterations in microbial community structure and reduced diversity at the genus and species levels. Consequently, fewer metabolic functions are available, increasing the risk of loss of host homeostasis (Figure 3) [60]. In contrast, higher alpha diversity is generally associated with improved intestinal homeostasis, reduced GI inflammation, and enhanced metabolic resilience [61].

### 4.1. Inflammatory and Compositional Mechanisms Linking Dysbiosis to Metabolic Disease

Multiple factors, including host genetics, age, intestinal diseases, geographical location, lifestyle habits, cigarette smoking, alcohol intake, sleep deprivation, and exposure to xenobiotic or environmental chemicals, can contribute to dysbiosis of the microbiota [62]. Among these, diet represents one of the most influential modulators of microbial composition and function.

High-fat diets have been shown to alter microbial composition not only through nutrient availability but also by increasing oxidative stress via reactive oxygen species (ROS) and by inducing epigenetic changes, which can further intensify microbial imbalance [63]. In mice, increased host-derived ROS production has been shown to modify gut microbiota composition and reduce microbial species diversity [64]. These diet-induced selective pressures reduce overall diversity and shift the relative abundance of specific taxa.

At the compositional level, distinct taxonomic alterations have been associated with metabolic disease. For instance, reduced abundance of *Bacteroides thetaiotaomicron* has been inversely correlated with circulating glutamate in obesity. In contrast, increased abundance of *Prevotella copri* and *Bacteroides vulgatus* has been associated with insulin resistance (Figure 3A). Similarly, depletion of butyrate-producing bacteria, including members of the *Clostridia* class, is linked to impaired barrier integrity and low-grade inflammation. In contrast, enrichment of *Proteobacteria* is associated with increased lipopolysaccharide burden and systemic inflammation [65].

Beyond changes in taxonomic composition, dysbiosis-associated inflammatory states are accompanied by alterations in microbial functional potential that reinforce metabolic dysfunction. Loss of barrier-supporting metabolites permits increased translocation of microbial components such as LPS, which activate innate immune receptors, including Toll-like receptors, and promote NF-κB signaling [66]. Although alterations in gut microbiota composition have been consistently associated with obesity-related metabolic disturbances, enrichment of taxa such as *Faecalibacterium*, *Bifidobacterium*, *Lactobacillus*, *Coprococcus*, and *Methanobrevibacter* is associated with a lower risk of developing metabolic diseases such as type 2 diabetes and insulin resistance. These taxa are frequently described as producers of anti-inflammatory metabolites linked to reduced inflammation and improved metabolic profiles [67,68]. In addition, these species are linked to hydrogen peroxide production, which can inhibit biofilm formation by some pathogenic species. Conversely, disruption of these communities is associated with compromised barrier function, enhanced inflammatory signaling, and increased susceptibility to metabolic disease (Figure 3B) [69].

Accordingly, gut microbiota diversity is widely considered a marker of intestinal and metabolic health. Reduced alpha diversity has been consistently associated with inflammatory gut disorders and metabolic dysfunction. Loss of butyrate-producing communities and expansion of pro-inflammatory taxa alter mucosal immune regulation, thereby linking microbial ecological imbalance and metabolic disease. At the same time, metabolic diseases such as type 2 diabetes and obesity, which are associated with reduced alpha diversity (Figure 3C), are characterized by altered microbial diversity patterns rather than uniform directional change, reflecting the complex interplay between microbial composition, inflammation, and metabolic regulation [70]. However, obesity and type 2 diabetes are not universally characterized by reduced alpha diversity. Large-scale multi-omics studies have shown that these conditions may instead be associated with specific functional alterations, including changes in carbohydrate metabolism, branched-chain amino acid biosynthesis, and bile acid transformation, even in the absence of major diversity loss [71,72].

### 4.2. Antibiotic Exposure as a Driver of Gut Microbiome Composition

Gut dysbiosis may arise from environmental factors, including diet, xenobiotics, and other exogenous compounds, as well as from antibiotic therapy, which disrupts microbial community structure and function. The impact on the host depends on the drug spectrum, administration, and host characteristics [73].

Unlike gradual diet-driven remodeling, antibiotic exposure represents an acute ecological disturbance that rapidly alters microbial community structure and reduces colonization resistance. Antibiotic exposure has been shown to deplete beneficial genera such as *Bifidobacterium* and *Faecalibacterium*, as well as to alter the relative abundance of the phylum *Firmicutes*. These changes disrupt gut homeostasis and contribute to the development and expansion of antimicrobial resistance [74]. Moreover, antibiotic-induced dysbiosis is associated with the depletion of key bacterial groups, including butyrate-producing members of the class *Clostridia,* and with increased susceptibility to opportunistic pathogens such as *Clostridioides difficile* [75].

Furthermore, in antibiotic-treated mice, reduced gut alpha diversity was associated with altered SCFA production, while systemic immune cell composition remained largely preserved under basal conditions. However, immune cells from antibiotic-treated hosts exhibited enhanced pro-inflammatory responses upon stimulation, suggesting that microbiota disruption primes the host immune system toward increased inflammatory susceptibility [76]. Given the central role of microbial imbalance in metabolic host health, strategies aimed at restoring microbial ecological stability have gained increasing attention. Studies have shown that consuming certain bacterial groups, including *Lactobacillus plantarum* and *Bifidobacterium,* can reduce the negative host effects associated with dysbiosis. In addition, exposure to certain xenobiotic compounds used to treat metabolic diseases, such as type 2 diabetes, has been linked to increased levels of butyrate-producing bacteria. For example, metformin has been shown to promote enrichment of these beneficial taxa, contributing to the restoration of a healthier microbial ecosystem [68].

### 4.3. Gut–Brain Axis Regulation of Metabolism

The gut–brain axis integrates a bidirectional communication network between the GI tract and the central nervous system through neural, endocrine, immune, and metabolic pathways. These signaling pathways are distinct yet interconnected, coordinating intestinal and central metabolic responses. The gut microbiome plays a central role in modulating this axis by producing metabolites and signaling molecules that influence appetite regulation, energy balance, and glucose homeostasis. Beyond the local inflammatory mechanisms described above, disruption of the gut microbiome can alter gut–brain communication by affecting microbial-derived metabolites, gut hormone secretion, and inflammatory signaling, thereby providing an additional mechanistic link between dysbiosis and the development of metabolic disease [77].

A key mechanism linking microbial metabolism to host metabolic control involves modulation of gut hormone secretion. Butyrate, propionate, and acetate act on enteroendocrine cells by binding to free fatty acid receptor 2 and 3 (FFAR2 and FFAR3) expressed on epithelial cells of the small intestine and colon, thereby stimulating the release of hormones such as glucagon-like peptide-1 (GLP-1) and peptide YY (PYY), which regulate satiety and glycemic control. These hormones transmit signals to the brain through systemic circulation and vagal afferent pathways [10,78].

Similarly, neural signaling pathways provide an additional layer of metabolic control; microbiota-driven modifications of host-derived molecules, such as bile acids, can influence vagal afferent signaling and hypothalamic circuits involved in appetite control and energy expenditure, providing a mechanistic basis for microbiome-associated changes in metabolic regulation [77].

Tryptophan, an essential amino acid, serves as a precursor for serotonin (5-HT), a neurotransmitter crucial for regulating GI physiology, including motility, secretion, and gut–brain signaling. While host enterochromaffin cells synthesize serotonin from tryptophan, a fraction of dietary tryptophan that the host does not absorb in the small intestine remains available in the intestinal lumen, where specific gut microorganisms can utilize it as a metabolic substrate. Through decarboxylation reactions, these bacteria convert tryptophan into tryptamine, which can activate serotonin receptor 4 (5-HT4R), thereby influencing intestinal motility and contributing to gut–brain communication, as colonization of germ-free mice with tryptamine-producing bacteria has been shown to enhance colonic secretion and improve gastrointestinal motility [79].

## 5. Dietary Intervention Strategies

The diversity of the gut microbiota partly determines the microbial response to dietary interventions; however, changes in food intake can alter its composition and function, thereby affecting host health (Table 2). Dietary phytochemicals are commonly found in plants such as pepper, *curcuma domestica*, *moringa oleifera,* and coffee. These compounds represent a broad group of natural compounds, including capsaicinoids, carotenoids, curcumin, polyphenols, flavonoids, tannins, and phenolic acids such as caffeic acid [80]. A small proportion of ingested phytochemicals is metabolized as xenobiotics and exhibits low bioavailability; however, the remaining compounds reach the colon, where intestinal bacteria modify their chemical structure through hydrolysis, reduction, dihydroxylation, demethylation, decarboxylation, and ring fission, leading to the production of bioactive metabolites [81]. For example, a study in mice revealed that the gut microbiota may influence the biotransformation of curcumin to dihydrocurcumin (DHC), tetrahydrocurcumin (THC), and hexahydrocurcumin (HHC), with implications for host health [82]. Dietary modifications, ranging from changes in specific plant-derived components to broader dietary patterns, together with microbiota-mediated transformations, can modulate gut microbial composition and function, thereby influencing host health.

Regular consumption of phytochemical-rich foods, including herbs, spices, fruits, legumes, and whole grains, as well as Mediterranean and green Mediterranean diets, has been associated with modulation of microbiota composition and function and with reductions in host diseases such as obesity and diabetes. Diets characterized by high intake of whole grains, prebiotics, fruits, and vegetables are associated with lower concentrations of quinolinic acid, an NMDA receptor agonist that exerts neurotoxic effects at elevated levels [92]. This dietary pattern is associated with increased abundance of *Faecalibacterium prausnitzii* and *Anaerobutyricum hallii,* both of which can produce butyrate. Moreover, this dietary pattern has also been associated with improved regulation of glucose metabolism and inflammatory markers [93].

In contrast, dietary interventions that restrict fermentable sugars, such as the ketogenic diet, are associated with distinct alterations in gut microbial composition. Individuals consuming diets characterized by high fat and protein intake and very low carbohydrate consumption reduce the availability of microbiota-accessible carbohydrates, leading to decreased abundance of fiber-degrading and short-chain fatty acid-producing bacteria [94]. These microbial shifts alter microbial metabolic output. Nevertheless, several studies have also associated ketogenic diets with potential benefits, including improved glucose regulation and effects on neuropsychiatric disorders.

Overall, specific dietary patterns, when combined with microbiota modulation, can regulate host health. In this context, the Mediterranean diet, which includes dietary fiber and antioxidant-rich bioactive compounds, has been shown to modulate the gut microbiota. Studies report increased levels of *Bacteroidetes*, *Clostridium*, *Faecalibacterium prausnitzii*, *Lactobacilli*, and *Bifidobacteria*, along with a reduced relative abundance of the phylum *Firmicutes* [95]. These microbial shifts influence the production of beneficial metabolites, which are associated with improved immune and metabolic health in the host.

## 6. Future Perspectives

Advances in elucidating how specific dietary patterns shape the gut microbiota are essential for clarifying the complexity of host metabolic regulation. Moving beyond descriptive associations toward a mechanistic characterization of the diet–microbiota–host interaction is particularly important for microbial biotransformation, which converts dietary phytochemicals into bioactive metabolites. Identifying the key microbial enzymes and consortia responsible for these processes will be pivotal to elucidating how gut-derived molecules function as signaling ligands that regulate glucose and lipid homeostasis, among other processes. Ultimately, integrating multi-omics approaches with host metabolic phenotyping will enable the development of precision nutrition strategies, allowing targeted dietary interventions to correct dysbiosis and mitigate the progression of metabolic disorders such as obesity and type 2 diabetes.

## 7. Conclusions

Collectively, current evidence indicates that dietary modulation of the gut microbiota is a key determinant of host metabolic regulation. By transforming dietary components into bioactive metabolites, the microbiome modulates intestinal barrier integrity, immune responses, and systemic metabolic homeostasis, thereby playing a central role in regulating host physiology. Alterations in microbiome composition and function are consistently associated with metabolic disorders and reflect both structural shifts in microbial communities and functional changes in microbial metabolic activity. In addition to regulating peripheral metabolic pathways, the gut microbiome contributes to host energy balance through the gut–brain axis by integrating microbial-derived metabolites with hormonal and neuroendocrine signaling. A deeper understanding of diet–microbiome–host interactions is essential for advancing integrative and translational strategies to prevent and manage microbiome-associated metabolic diseases.

## Figures and Tables

**Table 1:** Gut microbiota-derived metabolites and their roles in host metabolic regulation.

Microbial Metabolite	Microbial Producer	Metabolic-Host Effect	Reference
Acetate	*Akkermansia muciniphila*, *Bacteroides* spp., *Bifidobacterium* spp., *Prevotella* spp., *Ruminococcus* spp., *Clostridium* spp., *Streptococcus* spp.	Stimulates ^1^GLP-1/PYY release; reduces energy intake and adiposity	[30,31]
Propionate	*Bacteroides* spp., *Phascolarctobacterium succinatutens*, *Dialister* spp., *Veillonella* spp., *Megasphaera elsdenii*, *Coprococcus catus*, *Roseburia inulinivorans*, *Ruminococcus obeum*	Stimulates ^1^PYY/GLP-1 release; modulates hepatic glucose production; improves satiety and lipid metabolism	[30,32]
Butyrate	*Coprococcus comes*, *Coprococcus eutactus*, *Anaerostipes* spp., *Eubacterium hallii*, *Faecalibacterium prausnitzii*, *Roseburia* spp.	Enhances intestinal barrier function; exerts anti-inflammatory and insulin-sensitizing effects	[30,33]
Tryptamine	*Clostridium sporogenes*, *Ruminococcus gnavus*	Activates ^1^5-HT4 receptor; modulates gut motility; influences gut–brain signaling	[34]
^1^ CLA	*Bifidobacterium breve*, *Bifidobacterium animalis*, *Lactobacillus rhamnosus*, *Lactobacillus plantarum*	Anti-inflammatory effects	[35,36]

^1^ GLP-1, Glucagon-Like Peptide-1; PYY, Peptide YY; 5-HT4, 5-hydroxytryptamine; CLA, conjugated linoleic acids.

**Table 2:** Effects of dietary patterns on gut microbiota composition and microbiota-derived metabolic outputs.

Diet	Composition	Microbiome Effect	Study Model	Reference
Fiber-rich diet	* IDF:SDF = 1:9–9:1	High * IDF (≥80%): * ↑ α-diversity, * ↑ *Parabacteroides*/*Prevotella* (* TCA cycle). High * SDF (≥60%): * ↑ *Akkermansia*, * ↑ acetate/propionate (glycerophospholipid metabolism)	Mice	[83]
Animal-protein (Eggs, and beef)	-	* ↑ *Firmicutes* (Clostridiales); * ↓ diversity	Animal intervention	[84]
Animal-protein (Red meat-rich diet)	200 g/day (44 g protein)	Shifts in specific taxa (e.g., *Clostridium* spp.)	Human	[85]
Animal-protein (Beef protein)	~93% protein; higher methionine (1.75%); higher Lysine/Arginine ratio (1.25)	* ↑ *Firmicutes*	Hamster	[86]
Animal-protein (Pork protein)	~92% protein; methionine 1.05%; high sulfur amino acids	Microbiota-dependent cholesterol regulation	Hamster	[86]
Animal-protein (Chicken protein)	~92% protein; moderate methionine (1.65%)	Microbiota modulation of lipid metabolism	Hamster	[86]
Plant-derived protein (Soybean)	~94% protein; low methionine (0.66%); low Lysine/Arginine ratio (0.80)	* ↑ *Bacteroidetes*; improved lipid profile	Hamster	[86]
Plant-derived protein (Pea)	~93% protein; methionine 0.77%	Altered SCFA-producing taxa	Hamster	[86]
Plant-derived protein (Rice)	~94% protein; lowest sulfur amino acids (0.39)	Distinct microbial clustering	Hamster	[86]
High-fat diet	42% kJ fat; 0.2% cholesterol; isocaloric	Microbiota-dependent metabolic regulation	Mouse	[87]
Unsaturated fat-rich	37% kJ fat (21% * MUFA, 10% * PUFA, 6% * SFA); 48% carb; 15% protein; ~30 g fiber/8786.4 kJ	Microbiota-derived metabolites (circulating SCFAs)	Human	[88,89]
High-fat diet	60% kJ fat (lard)	* ↓ *Bacteroidetes*; * ↑ *Firmicutes*; * ↑ F/B ratio increases the proportion of * LPS-containing bacteria	Mice	[90]
Western diet	57% kJ fat (30% lard) + 0.2% cholesterol; 20% sucrose; fructose/glucose solution; 6 mole	* ↑ *Firmicutes*, *Proteobacteria*; * ↓ *Bacteroidetes*, *Fusobacteria*; * ↓ α-diversity	Male mice	[91]

* IDF, Insoluble dietary fiber; SDF, Soluble Dietary Fiber; TCA cycle, Tricarboxylic Acid cycle; MUFA, Monounsaturated Fatty Acids; PUFA, Polyunsaturated Fatty Acids; SFA, Saturated Fatty Acids; LPS, Lipopolysaccharide; ↑ increase; ↓ decrease; F/B ratio, *Firmicutes*/*Bacteroidetes*.

## Data Availability

No new data were created or analyzed in this study. Data sharing is not applicable to this article.
